# IL-1β As Mediator of Resolution That Reprograms Human Peripheral Monocytes toward a Suppressive Phenotype

**DOI:** 10.3389/fimmu.2017.00899

**Published:** 2017-08-03

**Authors:** Katharina Giesbrecht, Mariel-Esther Eberle, Sabine J. Wölfle, Delal Sahin, Aline Sähr, Valerie Oberhardt, Zach Menne, Konrad A. Bode, Klaus Heeg, Dagmar Hildebrand

**Affiliations:** ^1^Medical Microbiology and Hygiene, Centre for Infectious Diseases, University Hospital Heidelberg, Heidelberg, Germany; ^2^DZIF German Center for Infection Research, Brunswick, Germany

**Keywords:** resolution of inflammation, tolerogenic factors, signal transducer and activator of transcription 3, indoleamine 2,3-dioxygenase, PD-L1, adenosine, toll-like receptor-ligand, IL-1β

## Abstract

During infection pathogen-associated molecular patterns activate immune cells to initiate a cascade of reactions leading to inflammation and the activation of the adaptive immune response culminating in the elimination of foreign pathogens. However, shortly after activation of the host defense machinery, a return to homeostasis is preferred to prevent inflammation-induced tissue damage. This switch from the initial immunogenic to the subsequent tolerogenic phase after clearance of the infection can be mediated through highly plastic peripheral monocytes. Our studies reveal that an early encounter with toll-like receptor 7/8-ligand R848 mediates a strong pro-inflammatory monocytic phenotype that primes its own reprogramming toward an immunosuppressive one. Previously, we showed that these R848-treated antigen-presenting cells (APCs) fail to activate allogeneic T cells and induce regulatory T cells (Tregs) through signal transducer and activator of transcription 3 (STAT3)-dependent PD-L1. Here, we further demonstrate that R848-treated APCs suppress CD3/CD28-mediated and dendritic cell-mediated T cell activation and that adenosine and indoleamine 2,3-dioxygenase/kynurenin pathways are involved in tolerance induction. Reprogramming of monocytes after R848 stimulation requires the pro-inflammatory cytokine IL-1β and a boosted IL-6 release. The subsequent autocrine prolonged activation of STAT3 induces direct upregulation of tolerogenic factors which finally downregulate proliferation of activated T cells and mediate Tregs. Thereby our study suggests that inflammatory cytokines, such as IL-1β and IL-6, should be considered as mediators of resolution of inflammation.

## Introduction

Our sophisticated immune system is perpetually searching danger signals to recognize and eliminate invading pathogens. Blood monocytes serve as the first line of host defense and are equipped with pattern recognition receptors (PRRs), such as toll-like receptors (TLRs) and NOD-like receptors (NLRs), to recognize and respond to infection-associated pathogen-associated molecular patterns (1–3). The triggered inflammatory response subsequently activates adaptive immunity to effectively protect the host and establish a memory immune response. Inflammation is required for clearance of infection. Nevertheless, excessive inflammation is energetically expensive and, unchecked, could harm the host. Therefore, timely resolution of inflammation is essential. The resolution of immune responses and restoration of homeostasis are not just achieved through catabolism of pro-inflammatory factors and fading of the response, but also through active regulatory processes that are initiated by the inflammation itself (2, 4). Although neutrophils and macrophages are conventionally considered as major cell types during the resolution phase, the role of accessory cells, such as regulatory T cells (Tregs) and monocytic myeloid-derived suppressor cells (mo-MDSCs), have more recently become apparent (2, 4). Due to their high plasticity, peripheral monocytes are best suited to develop a phenotype that triggers inflammation or mediates the shift to resolution. Strongly pro-inflammatory at the beginning of infection, blood monocytes can be reprogrammed from an activating to a suppressive phenotype. Thereby, the cells show a shifted rather than a shutdown gene expression and mediate a transition from inflammation to tolerance (5, 6). One key regulator of tolerance is signal transducer and activator of transcription 3 (STAT3) (7, 8). As a cytokine receptor-activated transcription factor, STAT3 mediates the expression of several tolerogenic factors and is known to mediate generation of monocytes with suppressive character in distinct cancer types (9, 10). A similar occurrence involving STAT3-mediated upregulation of resolution factors can be observed *in vitro* when exposing human peripheral monocytes to a TLR-ligand (LPS, R848) early during GM-CSF/IL-4-driven differentiation to immature dendritic cells (iDCs) (11). Recently, we have shown that the generated myeloid phenotype, which retains CD14 and downregulates HLA-DR and CD1a, releases very high amounts of pro-inflammatory IL-6 through TLR-mediated MAPkinase activation. However, through a prolonged autocrine activation of STAT3 and subsequent PD-L1 expression, the cells eventually fail to activate CD4^+^ T cells and instead induce Tregs (11). In our current study, we aimed to further analyze the inflammation-mediated reprogramming of human peripheral monocytes after TLR activation. We identified the TLR7/8 ligand R848 as the most potent activator of monocytes. R848-treated antigen-presenting cells (APCs) revealed a strong inflammatory phenotype with respect to TNFα, IL-12p40, IL-6, and IL-1β release. IL-1β-boosted IL-6 production and the subsequent STAT3 hyper-activation then mediate the upregulation of several immune-suppressive factors. Thereby the combination of indoleamine 2,3-dioxygenase (IDO)-generated kynurenine, PD-L1, and adenosine (ADO) potently mediates suppression of CD3/CD28-activated T cells. These data show that a PRR-mediated inflammatory response primes its own resolution, through induction of distinct tolerogenic factors and pathways. Thereby our study supports the hypothesis that hyper-inflammation reprograms peripheral monocytes to a regulatory phenotype with immunosuppressive properties.

## Materials and Methods

### Reagents

Recombinant human IL-4 and GM-CSF were purchased from R&D Systems (Wiesbaden, Germany). LPS from Salmonella Minnesota (smooth form) was provided by U. Seydel (Borstel, Germany), R848 (Resiquimod, imidazoquinoline compound) was from ALEXIS (Lausen, Switzerland). Poly I:C (PIC) was obtained from InvivoGen (San Diego, CA, USA). IDO inhibitor 1-methyl-tryptophan (1MT), ADO-receptor antagonist ZM-241385 and ADO from Sigma-Aldrich (Taufkirchen, Germany), Calprotectin from Hycult Biotech (Uden, Netherlands), kynurenine and LILRA3 from Novoprotein (Summit, USA), α-IL-1β (eBioscience, Frankfurt/Main, Germany).

### Isolation of Cells

PBMCs were isolated from fresh blood or buffy coat from healthy donors by means of density gradient centrifugation (Biocoll separating solution, 1.077 g/ml, Biochrom AG, Berlin, Germany). PBMCs were washed three times with PBS and CD14^+^ magnetically labeled cells were positively selected *via* the autoMACS separator (autoMACS, program: possel, Miltenyi Biotec, Bergisch Gladbach, Germany) twice. Untouched CD4^+^ T cells were purified *via* the CD4^+^ T Cell Isolation Kit (MiltenyiBiotec) and autoMACS program: depletes. Cells were cultured in RPMI 1640 (Sigma-Aldrich, Taufkirchen, Germany) supplemented with 100 IU/ml of penicillin, 100 µg/ml streptomycin containing 10% heat inactivated fetal calf serum (Promocell, Heidelberg, Germany) at 37°C in a humidified atmosphere in the presence of 5% CO_2_.

### Differentiation and Stimulation of Cells

2 × 10^6^ sorted CD14^+^ monocytes were plated in 24-well plate format in 2 ml. To obtain iDCs, cells were supplemented with 10 ng/ml rhGM-CSF and 20 ng/ml rhIL-4. TLR-ligand-treated APCs were generated by the addition of 1 µg/ml R848 (R848-treated APCs), 30 ng/ml LPS (LPS-treated APCs), or 50 µg/ml PIC (PIC-treated APCs). Cells were harvested after different time-points as indicated, respectively. For maturation [mature DCs (mDCs)], iDCs were stimulated on day 6 for an additional 18 h with 30 ng/ml LPS. For inhibitor experiments, cells were treated with 240 µM 1MT (1 h prior to stimulation and again after 2 days), 10 µg/ml anti-PD-L1, and 10 µM ZM-241385 (1 h prior to stim.).

### Mixed Lymphocyte Reaction (MLR)

Mixed lymphocyte reactions were performed in allogeneic settings: purified 1 × 10^5^ CD4^+^ T cells (CD4^+^ T Cell Isolation Kit, MiltenyiBiotec) and 1 × 10^4^ monocytes (Mitomycin C-pre-treated) were cocultured. When indicated T cells were activated with anti-CD3 and anti-CD28-coated beads (T Cell Activation/Expansion Kit, Miltenyi Biotec) at a ratio of 1:2. Cells were cultured for 4 days and exposed to [3H]-thymidine (Amersham Pharmacia Biotech GmbH, Freiburg) during the last 6 h of culture. Thymidine uptake was measured by using a liquid scintillation counter.

### Carboxyfluorescein Succinimidyl Ester (CFSE)-Proliferation Assay

Monocytes were treated as indicated. After 3 days, allogeneic, CFSE-labeled, CD3/CD28-activated CD4^+^ T cells were added in a ratio of 1:2. For CFSE-labeling CD4^+^ T cells were incubated 10 min at r. t. in 0.3 mM CFSE/PBS (MolecularProbes, San Diego, CA, USA) and thereafter intensively washed. For determination of R848 APC-mediated suppression of iDC-mediated T cell proliferation 250,000 iDCs/ml were cocultured with 500,000 CFSE-labeled CD4^+^ T cells/ml. Increasing numbers of R848-treated APCs were added to the culture (50,000, 100,000, and 200,000 R848 APCs/ml). For determination of IL-6 dependency GM-CSF/IL-4 stimulated monocytes were treated with 1,000 ng/ml recombinant IL-1β (PeproTech, Hamburg, Germany) ± anti-IL-6 antibody (eBioscience, Frankfurt/main, Germany). After 3 days, cocultures were started with CFSE-labeled T cells. Three days after start of coculture, cell divisions were analyzed by determining the FITC signal using a FACScanto.

### Flow Cytometry

Three to five days after stimulation monocytes were analyzed for surface markers with antibody staining: α-CD1a-FITC, α-CD14-PE, α-CD16-PE-CY5 (BD Biosciences, Heidelberg, Germany), α-PD-L1-PE, α-CD85J (LILRB1)-APC, α-CD85K (LILRB4)-APC, and α-CD85D (LILRB2)-PE (eBioscience, Frankfurt/Main, Germany). Mean fluorescence was recorded using the FACS DIVA V 4.12 software on a FACS Canto (BD Biosciences). Overlays were performed with the Weasel v2.5 software (WEHI, Melbourne, VIC, Australia). FoxP3 expression in T cells was assessed using an anti-human FoxP3 Staining Kit (e-Biosciences, San Diego, CA, USA).

### Determination of IDO Activity

For the detection of the enzymatic activity of IDO, kynurenine was measured in the supernatants of iDCs and TLR-ligand-treated APCS after 3 days of culture. The protocol was adapted to the manufacturer’s instruction (Universität Ulm, Transplantationsforschung, Ulm, Germany). In brief, 150 µl cell culture supernatant was supplemented with 100 µl Trichloric acid (30%) for protein precipitation. After centrifugation, supernatant was transferred into 96-well plate format and incubated at 50°C for 30 min. For the measurement of kynurenine, a respective standard was used with the highest concentration of 50 µM, diluted stepwise 1:2. 150 µl color reagent was added to each well to start the reaction. After 5 min, absorbance was measured at 492 nm with a reference wavelength of 690 nm using a photometer (SUNRISE Absorbance reader, Tecan, Salzburg, Austria). Kynurenine concentrations were calculated with the Magellan V 5.0 software (Tecan, Salzburg, Austria).

### Enzyme-Linked Immunosorbent Assay (ELISA)

Cell-free supernatants were harvested and analyzed for IL-1β, IL-6, IL-12 (p40), IL-10, TNFα (BD Biosciences), LILRA3 (Cusabio), and HLA-G (Wuhan EIAab Science) by ELISA kits.

### Western Blotting

4 × 10^6^ cells were lysed in RIPA lysis buffer containing protease inhibitors (cOmplete™, Roche, Mannheim, Germany) and phosphatase inhibitor (PhosSTOP™, Roche). Subsequently, lysates were separated by 10% SDS-PAGE and electrotransferred to nitrocellulose membranes (Whatman Protran nitrocellulose membrane; neoLab, Heidelberg, Germany). After blocking (TBS/0.05% Tween-20/5% BSA) and washing (TBS/0.05% Tween) steps, immunoblotting with antibodies against adenosine deaminase (ADA) (abcam), arylhydrocarbon receptor (AhR), and IDO (Cell Signaling Technology) was performed. Analyzing HPRT (abcam), β-Actin, or GAPDH (Cell Signaling Technology) reassured equal loading. Detection was enhanced by chemiluminescence (ECL; Perkin Elmer, Groningen, Netherlands).

### RNA Purification and Quantitative RT PCR

4 × 10^6^ cells were harvested and subsequently total RNA was isolated using the High Pure RNA Isolation Kit (Roche, Mannheim, Germany). The first strand cDNA kit by Thermo Scientific (Waltham, MA, USA) was used for the synthesis of cDNA from equal amounts of RNA. ABsoluteTM qPCR SYBR^®^Green Low ROX Mix (Thermo Scientific, Waltham, MA, USA) was used to perform quantitative real-time PCR. For the calculation of relative expression, normalization to β-Actin mRNA expression levels as 2-ΔCt was done. All primers were synthesized by Eurofins MWG Operon (Ebersberg, Germany). Human β-Actin, sense, 5′-aga gct acg agc tgc ctg ac-3′, antisense, 5′-agc act gtg ttg gcg tac ag-3′, human IDO, sense, 5′-tta gag tca aat ccc tca gtc c-3′, antisense, 5′-ttt gca gat ggt agc tcc tc-3′, human ADA, sense, 5′-gac ctg gct gga gat gag ac-3′, antisense, 5′-gtc ttc cag ggt gtg gta gc-3′, human ENTPD1 (CD39), sense, 5′-agt tct gtg ctc agc ctt gg-3′, antisense, 5′-ttg cag aag gag gga gag aa-3′, human NT5E (CD73), sense, 5′-ggg agg aca ctc caa cac at-3′, antisense, 5′-agg cct gga cta cag gaa cc-3′, human S100A8, sense, 5′-atg ccg tct aca ggg atg ac-3′, antisense, 5′-acg ccc atc ttt atc acc ag-3′, S100A9 sense, 5′-tca tca aca cct tcc acc aa-3′, antisense, 5′-gtg tcc agg tcc tcc atg at-3′.

### Gene Array

Expression profiling of APCs was performed as follows. TLR-ligand-treated APCs and iDCs were generated as mentioned above (see Differentiation and Stimulation of Cells). 4 × 10^6^ cells were seeded in 24-well plates and used in three replicates. After 6 days, cells were harvested, lysed, and total RNA was isolated by means of High Pure RNA isolation kit (Roche, Mannheim, Germany). At least 500 ng of purified RNA (RIN-value ≥ 8.0) was utilized for gene array purposes. RNA purity and concentration was determined by means of RNA gel analysis using the Nano 2100 bioanalyzer, version 2.6 (DE54700489, Agilent Technologies, Berlin, Germany). RNA was used to synthesize cDNA, followed by *in vitro* transcription and labeling to produce Biotin-labeled cRNA, according to MessageAmp II aRNA Amplification kit (Ambion, Austin, TX, USA). Biotin-16-UPT was purchased from Roche Applied Sciences (Penzberg, Germany). The TotalPrep RNA Amplification Kit was used to column-purify the cRNA followed by quantification and determination of purity (RNA gel analysis, Nano 2100 bioanalyzer, Agilent Technologies). Expression profiling was performed by DKFZ-Genomics and Proteomics Core Facility (TP3, DKFZ, Heidelberg, Germany^1^). Labeled cRNA was hybridized to human HT-12 V4 Expression BeadChip and Microarray was scanned using a Beadstation array scanner. Data were quantile normalized and statistically analyzed (Benjamini–Hochberg) using Chipster analysis software.^2^ Only data-sets with a *p*-value ≤0.05 were examined as statistically significant and probes with a fold-change ≥2 and ≤0.5 were further analyzed. Raw data and quantile-normalized data are stored in NCBI’s Gene Expression Omnibus (GEO). They are accessible through GEO Series accession number GSE98480.^3^

### Statistical Analysis

The comparison of two data groups were analyzed by Mann–Whitney *U* test.

### Ethical Statement

This study was carried out in accordance with the recommendations of the ethics committee of the Medizinische Fakultät Heidelberg with written informed consent from all subjects. All subjects gave written informed consent in accordance with the Declaration of Helsinki. The study (taking of blood samples from healthy donors and treatment of blood leukocytes with microbial stimuli) was reviewed and approved by the ethics committee of Medizinische Fakultät Heidelberg.

## Results

**Figure 1:**
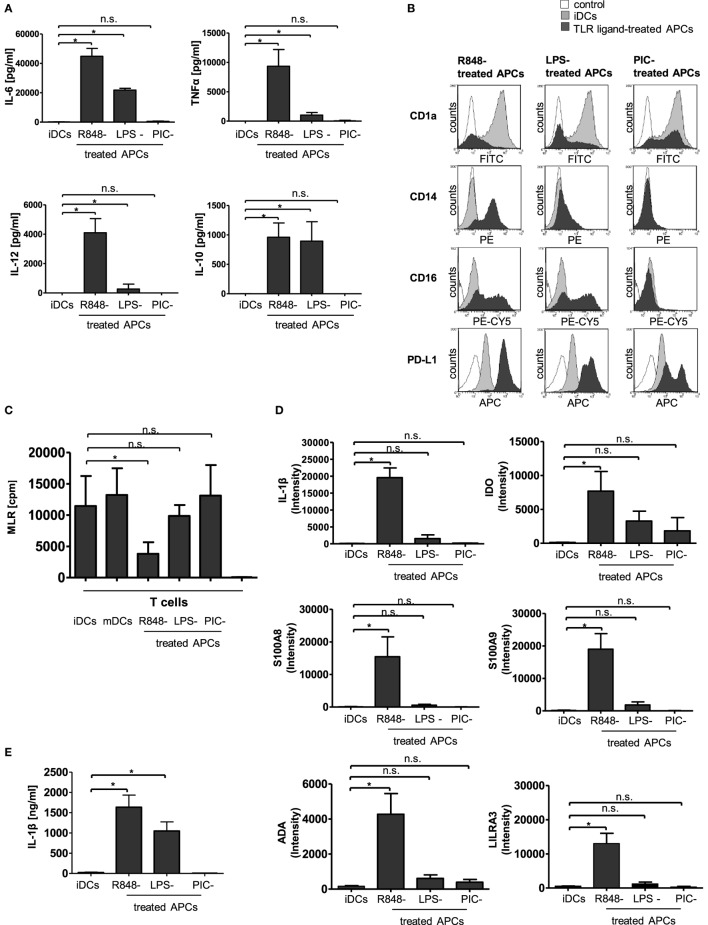
Characterization of toll-like receptor (TLR)-ligand-treated antigen-presenting cells (APCs). Isolated CD14^+^ monocytes were stimulated with GM-CSF and IL-4 [immature dendritic cells (iDCs)] or GM-CSF, IL-4, and R848 (R848-treated APCs) or LPS (LPS-treated APCs) or poly I:C (PIC-treated cells) for 3 days. **(A)** Supernatants of cultures were tested by enzyme-linked immunosorbent assay (ELISA) for release of IL-6, TNFα, IL-12p40, and IL-10. **(B)** Expression of surface markers CD1a, CD14, CD16, and PD-L1 was measured by staining with fluorescently labeled antibodies and flow cytometry. **(C)** Proliferation of responder CD4^+^ T cells was measured by [3H]-thymidine uptake after 96 h of stimulation with allogeneic iDCs, mature DCs (mDCs), R848-treated APCs, LPS-treated APCs, or PIC-treated APCs or responder cells alone (nk). **(D)** mRNA abundance. iDCs, R848-treated APCs, LPS-treated APCs, and PIC-treated APCs were analyzed by gene array. Shown is the intensity (abundance of probe-target hybridization) of selected highly upregulated genes. **(E)** IL-1β ELISA of culture supernatants. **(A,C)** show the respective mean values of four **(D,E)** of three individual experiments. *p ≤ 0.05 by Mann–Whitney *U* test. **(B)** are representative for three independent analyses. ELISA and mixed lymphocyte reaction (MLR) experiments are performed in duplicates.

In our previous work, it could be shown that an early encounter with R848 (Resiquimod, a low molecular weight synthetic molecule that activates immune cells *via* the TLR7/TLR8 MyD88-dependent signaling pathway) interferes with GM-CSF and IL-4-driven differentiation of human peripheral monocytes into iDCs (11). The generated R848-treated APCs present an atypical phenotype characterized as highly pro-inflammatory, yet failing to activate T cells and subsequently meditating immunosuppression through the induction of Tregs (11). In our current study, we aimed to clarify the connection between the hyper-inflammatory and immunosuppressive phase of R848-treated APC differentiation and further characterize the tolerogenic phenotype of R848-treated APCs. To find out whether the deviated differentiation is a general TLR-mediated mechanism, we first investigated the effect of three different TLR ligands, namely R848, LPS (TLR4 ligand), and PIC. PIC is structurally similar to double-stranded RNA present in some viruses and a demonstrated ligand for TLR3 (12). First, we established a cytokine profile of the TLR-ligand-treated APCs. In agreement with our previous findings (11), R848-treated APCs released high amounts of pro-inflammatory TNFα, IL-12p40, and, most pronounced, IL-6. In addition, immunosuppressive IL-10 was strongly produced. Cells treated with LPS released IL-6, TNFα, and IL-12p40 as well, albeit in much lower amounts. The LPS-treated APCs also released high amounts of IL-10. Treatment with PIC, however, did not result in an increase in any of the assayed cytokines (Figure 1A). Next, we checked the surface expression of CD14 and CD1a as differentiation markers. A hallmark of R848-treated APCs is the preservation of CD14 and loss of antigen-presenting compartment CD1a (11). LPS stimulation induced a less pronounced phenotype with diminished CD14 expression and a diversity of CD1a^−^ and CD1a^+^ cells. PIC-treated APCs downregulated CD14 and partially expressed CD1a (Figure 1B). In addition, we stained the cells with an anti-CD16-specific antibody. In addition to CD14, CD16 (FcγRIII) is often utilized to distinguish subsets of human monocytes (13, 14). R848-treated APCs and LPS-treated APCs both upregulated CD16 on their surface compared to iDCs. PIC stimulation did not induce an upregulation of FcγRIII (Figure 1B). PD-L1, which partly mediated Treg induction by R848-treated APCs (11), was also strongly upregulated in LPS-treated cells. PIC stimulation only marginally induced PD-L1 expression (Figure 1B). The subsequently performed MLRs show as expected that mDCs as well as iDCs mediate T cell proliferation. Furthermore, the MLR reveals that PIC-treated APCs lead to strong T cell proliferation similar to mDCs. LPS-treated APCs were still able to induce T-cell proliferation. R848-treated APCs failed to activate T cells (Figure 1C).

To characterize TLR-ligand-treated APCs in more detail, we performed an expression profile of iDCs cells in comparison to R848-treated APCs, LPS-treated APCs, and PIC-treated APCs. Raw data and quantile-normalized data are stored in NCBI’s GEO. They are accessible through GEO Series accession number GSE98480 (see text footnote 3). Within the upregulated genes, we identified a variety of interesting candidates with proposed impact on immunological tolerance, including IDO, S100A8 and S100A9, LILRA3 as a member of the leukocyte immunoglobulin-like receptors (LILR) family (also termed immunoglobulin-like transcript ILT family), and ADAa member of the ADO pathway (Figure 1D). Of these candidate genes, all were most potently induced by R848. LPS stimulation resulted in a much weaker induction than R848, while PIC only upregulated IDO and ADA in small amounts. Notably, the strongest upregulated gene in R848-treated APCs was pro-IL-1β (Figure 1D). 2-D Fluorescence difference gel electrophoresis confirmed this result for R848-treated APCs (Figure S1 in Supplementary Material). Furthermore, R848 induced not only a massive transcription of the IL-1β-precursor (Figure 1D) but also triggered release of high amounts of activated IL-1β (Figure 1E). iDCs and PIC-treated APCs did not induce any production of mature IL-1β, whereas LPS-treated APCs released detectable but limited amounts of IL-1β.

**Figure 2:**
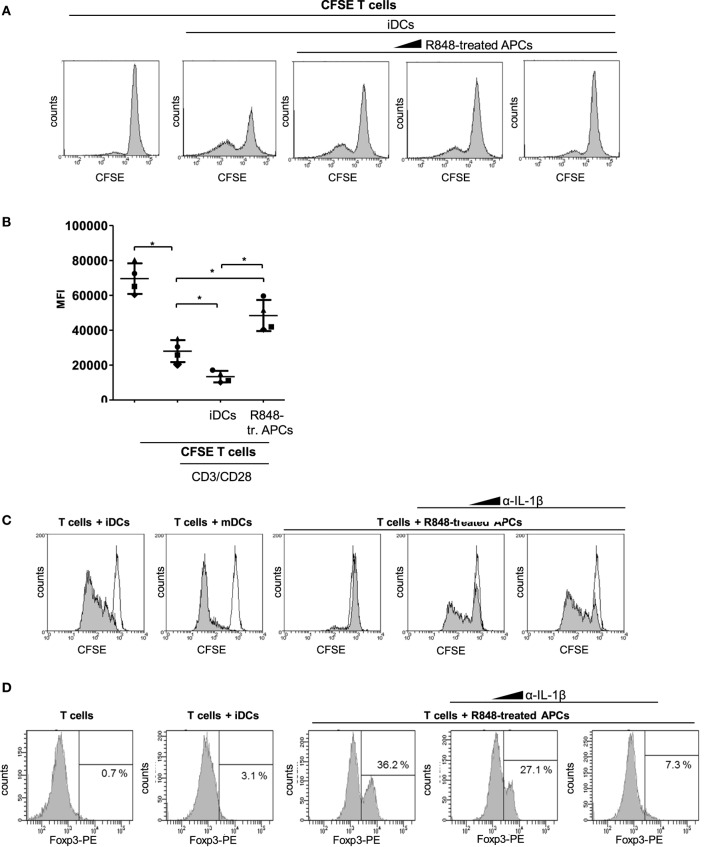
R848-treated antigen-presenting cell (APC)-mediated T cell suppression. **(A)** Carboxyfluorescein succinimidyl ester (CFSE)-labeled CD4^+^ T cells were cocultured for 3 days with immature dendritic cells (iDCs) with increasing amounts of R848-treated APCs in the culture. After 3 days, cell divisions were analyzed by determining the FITC signal using a FACScanto. **(B)** CFSE-labeled CD4^+^ T cells were activated with anti-CD3/CD28-coated beads and cocultured with either iDCs or R848-treated APCs. Additionally proliferation (CFSE signal) of not activated T cells was determined. MFl, mean fluorescence. **(C)** CD14^+^ monocytes were stimulated with GM-CSF and IL-4 (iDCs) or GM-CSF, IL-4, and R848 (R848-treated APCs) for 3 days. α-IL-1β antibody (300, 3,000 ng/ml) was added from the beginning of R848-treated APC differentiation. iDCs, mature DCs (mDCs), or R848-treated APCs ± α-IL-1β were added to allogeneic CFSE-labeled, activated CD4^+^ T cells. Loss of CFSE was measured after 5 days by flow cytometry. **(D)** CD4^+^ T cells were cocultured with iDCs or R848-treated APCs ± α-IL-1β antibody (300, 3,000 ng/ml). After 5 days T cells were intracellularly stained with a PE-labeled α-Foxp3-specific antibody and analyzed by a FACScanto for PE-positive cells in the lymphocyte population. Panels **(A,C,D)** show one representative result out of three experiments. Overlays were produced by Weasel.jar software. Panel **(B)** depicts mean values and SD of three individual experiments. *p ≤ 0.05 by Mann–Whitney *U* test.

In the following, we aimed to clarify whether R848-treated APCs just fail to activate T cells or whether they act suppressive on activated T cells. Therefore, we performed a coculture experiment with iDCs and CFSE-labeled T cells in which we added increasing amounts of R848-treated APCs. Figure 2A shows the iDC-mediated activation of T cells, which can be observed through a decreasing CFSE signal of proliferating cells. Furthermore the flow cytometry data reveal that the iDC-mediated activation is increasingly inhibited through increasing amounts of R848-treated APCs. To enhance the clone size of activated T cells in allogeneic coculture, we mimicked the iDC-mediated activation of T cells through stimulation with anti-CD3/CD28-coated beads. Figure 2B shows that R848-treated APCs also inhibit the CD3/CD28-boosted T cell proliferation very pronounced which additionally confirmed the suppressive character of R848-treated APCs. Next, we aimed to discover how the pro-inflammatory phenotype of R848-treated APCs in terms of cytokine release fits to the mediated T cell suppression. IL-1β is one of the most potent mediators of inflammation (15) but is also associated with downregulation of the immune system and immune defects (16, 17). So in the following experiments, we investigated whether the high release of IL-1β and the subsequent autocrine IL-1-receptor (IL-1R) signaling could mediate reprogramming of cells toward a resolving phenotype. To this effect, we treated blood monocytes right from the beginning of differentiation with an anti-IL-1β-specific antibody. After 3 days of differentiation, a MLR with CFSE-labeled CD3/CD28-activated CD4^+^ T cells was started. Figure 2C shows, as expected, that mDCs mediate the decrease of fluorescence implying robust T-cell proliferation. Alternatively, iDCs exhibited a smaller decrease in fluorescence and R848-treated APCs showed almost no activation of cocultured CD3/CD28-activated T cells. Inhibition of IL-1R signaling during differentiation of R848-treated APCs could restore the activating ability of the cells to induce T cell proliferation. According to our previously published data, R848-treated APCs mediate expansion of Tregs data (11). This expansion could be reduced with 300 ng/ml and abrogated with 3,000 ng/ml of anti-IL-1β-specific antibody (Figure 2D).

**Figure 3:**
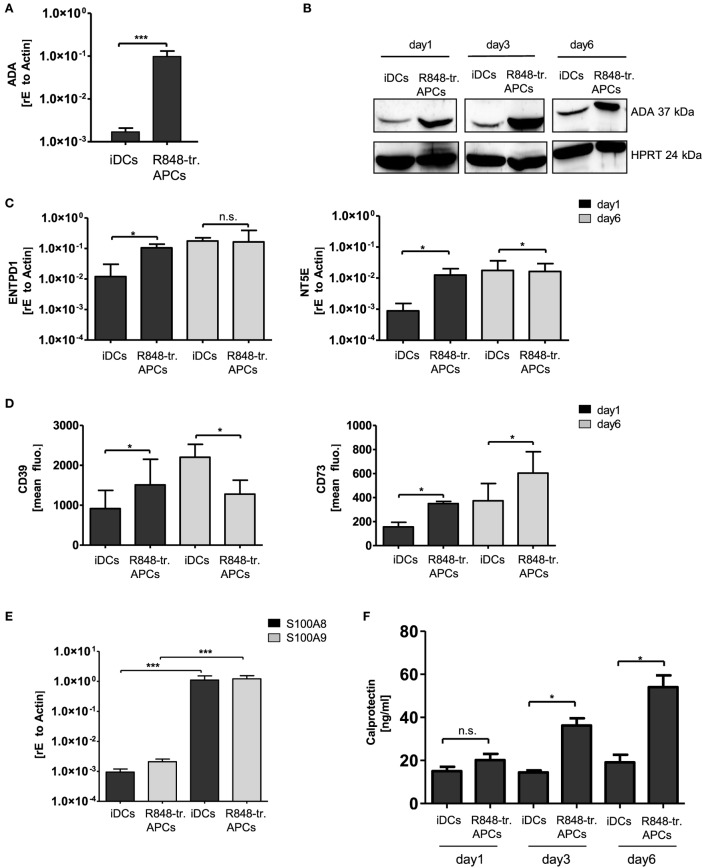
Adenosine deaminase (ADA) and S100A8/A9 induction in R848-treated antigen-presenting cells (APCs). Isolated CD14^+^ monocytes were stimulated with GM-CSF and IL-4 [immature dendritic cells (iDCs)] or GM-CSF, IL-4 and R848 (R848-tr. APCs) for 3 days. **(A)** RNA was isolated and cDNA produced. Induction of ADA was quantified by quantitative (q) RT PCR using sequence-specific primer (β-Actin as housekeeping gene) and SYBR green master mix. **(B)** For western blot analysis equal amounts of protein lysates were blotted and probed with antibodies against ADA and HPRT (loading control). **(C)** RT PCR analysis. Left-hand side: induction of ENTPD1 (gene encoding CD39); right-hand side: NT5E (gene encoding CD73) of 3 or 6 days treated iDCs and R848-treated APCs. **(D)** Flow cytometry analysis of CD39 (left graph) and CD73 (right graph). **(E)** RT PCR analysis of S100A8/A9 **(F)** enzyme-linked immunosorbent assay (ELISA) results of released S100A8/A9 heterodimer (calprotectin). **(A,C,D,F)** Mean values and SD of three individual experiments. **(E)** *n* = 5 *p ≤ 0.05 by Mann–Whitney *U* test. **(B)** One representative result out of three experiments. ELISA and RT PCR experiments are performed in duplicates.

In the next experiments, we verified further differentially regulated genes of the gene array and their possible contribution to the observed tolerogenic phenotype. As R848-treated APCs exhibited the most pronounced suppressive phenotype, we focused on genes upregulated in these APCs. One hit of the gene array was ADO-regulating enzyme ADA, which is part of the anti-inflammatory ADO signaling cascade. ADO is a metabolite of extracellular ATP and inhibits immune cell activation through binding to its receptor and subsequent regulation of intracellular cAMP (18, 19). To investigate a possible impact of ADO in our system, we first verified the high expression of ADA by RT PCR (Figure 3A). In addition, Western blotting confirmed the abundance on protein level for at least 6 days of culturing (Figure 3B). To further investigate the impact of the ADO pathway, we then checked the expression of the ADO-generating enzymes CD39 (nucleoside triphosphate dephosphorylase) and CD73 (ecto-5′-nucleotidase). Both RT PCR and flow cytometry data revealed that the two enzymes are differentially regulated. CD39 (gene name *ENTPD1*) which catalyzes the first step in ATP break down was induced only within the first day. CD73 (gene name *NT5E*), which accomplishes the final dephosphorylation step, was highly expressed for a longer period of 6 days (Figures 3C,D). These results suggest that essential parts of the ADO pathway are upregulated in R848-treated APCs.

Other genes of interest showing upregulation in the gene array were S100A8 and S100A9. Both proteins belong to the family of alarmins, and can be induced in various cell types through IL-6-mediated STAT3 activation (20). The proteins tend to form dimers such as the heterodimer S100A8/A9 (calprotectin) *via* the two calcium-binding loops in their structures. Partly diverging immune functions have been described for calprotectin, including pro- and anti-inflammatory properties (4). Our RT PCR analysis verified the gene array data (Figure 3E) and ELISA analysis confirmed that R848-treated APCs release higher amounts of calprotectin than iDCs (Figure 3F).

**Figure 4:**
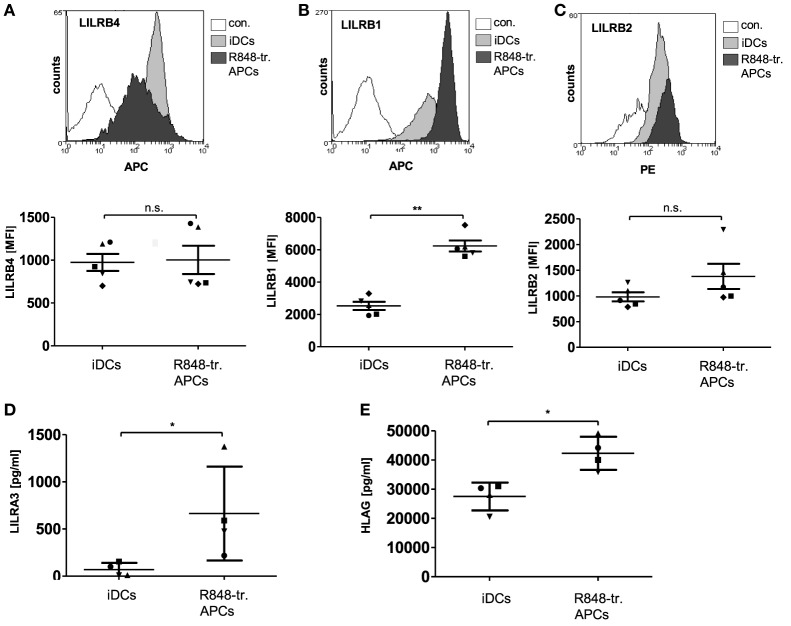
Leukocyte immunoglobulin-like receptor (LILR) expression in R848-treated antigen-presenting cells (APCs). Immature dendritic cells (iDCs) and R848-treated APCs were analyzed with fluorescently labeled antibodies by flow cytometry for surface expression of **(A)** LILRB4, **(B)** LILRB1, and **(C)** LILRB2. Top rows show one representative overlay of results, lower row shows according quantification of five donors. **(D)** LILRA3-enzyme-linked immunosorbent assay (ELISA) **(E)** HLAG-ELISA. **(A–E)** One symbol represents one donor. Statistic: *p ≤ 0.05 by Mann–Whitney *U* test. ELISAs are performed in duplicates.

In addition, the LILRs protein family appeared to be upregulated in R848-treated APCs based on the gene array results. Interestingly, LILRs have been shown to have a deleterious role in cancers and beneficial effects in transplantation (21). The LILRs are a family of innate immune receptors that can be divided into activating (LILRA1-2, 4–6), inhibitory (LILRB1-5), and the less characterized soluble (LILRA3) members (21). Analysis of the gene array data revealed that some inhibitory family members were upregulated in R848-treated APCs, with the most pronounced difference in LILRA3 expression (Figure 1D). So we next verified the data on the protein level and found LILRB4 surface expression to be variable between different donors (Figure 4A), while LILRB1 (Figure 4B) and LILRB2 (Figure 4C) were consistently upregulated in R848-treated APCs. ELISA analyses confirmed the robust release of soluble LILRA3 in R848-treated APCs compared to no detectable LILRA3 in iDC supernatants (Figure 4D). LILRA3 might act as an antagonist for activating LILRs, whereas LILRB1 and LILRB2 transduce their inhibitory effect upon binding their high affinity ligand HLA-G. Indeed, ELISA analysis showed that the supernatants of R848-treated APCs contained significant amounts of HLA-G (Figure 4E).

**Figure 5:**
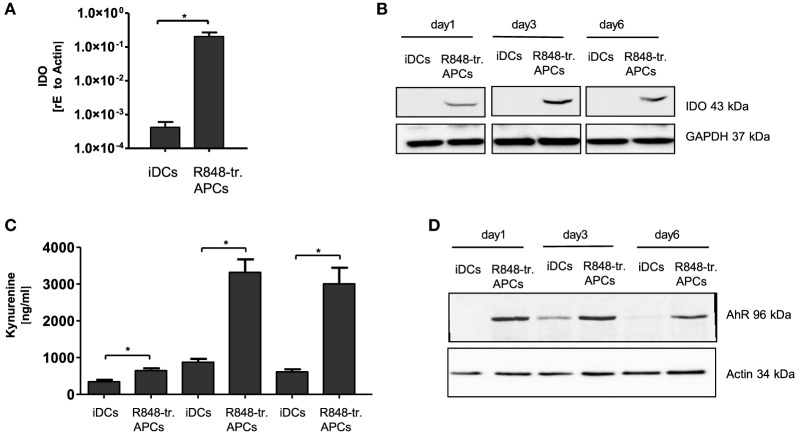
Indoleamine 2,3-dioxygenase (IDO) production in R848-treated antigen-presenting cells (APCs). Isolated CD14^+^ monocytes were stimulated with GM-CSF and IL-4 [immature dendritic cells (iDCs)] or GM-CSF, IL-4 and R848 (R848-treated APCs) for the indicated time course. **(A)** RNA was isolated and cDNA produced. Induction of IDO was determined by RT PCR using sequence-specific primer and SYBR Green Master mix. Results were normalized against β-Actin. **(B)** For western blot analysis, equal amounts of protein lysates were blotted (GAPDH as loading control) and probed with antibodies against IDO. **(C)** Kynurenine (IDO metabolite) quantification in culture supernatant. **(D)** Expression analyses of arylhydrocarbon receptor (AhR) by western blots. **(A,C)** show mean and SD of three donors. Experiments performed in duplicates. Statistic: *p ≤ 0.05 by Mann–Whitney *U* test. Western blots were repeated three **(B)** or two **(D)** times with comparable results.

The fourth potentially tolerance-mediating mechanism we investigated was the IDO-kynurenine pathway. IDO catalyzes the first and rate-limiting step of tryptophan catabolism to *N*-formyl-kynurenin, which can inhibit T cell activation and induce Tregs (22, 23). Our experiments in R848-treated APCs clearly confirmed the high induction of *IDO1* gene transcription predicted from the gene array (Figure 5A). Furthermore, the IDO protein was stably present in R848-treated APCs from day 1 till day 6 of culture, while IDO was not detectable in iDCs on any day of culture (Figure 5B). The question of whether the expressed enzyme actually catalyzes the degradation of tryptophan in our system was addressed using a kynurenine detection assay. The supernatant of R848-treated APC cultures contained high amounts of kynurenine from day 3 on to day 6 (Figure 5C). Kynurenine is known to bind and activate the AhR. The ligand-activated transcription factor AhR can promote Treg cell differentiation (22, 24) and also align APCs toward a tolerogenic phenotype (25, 26). Interestingly, STAT3 can bind to the AhR promoter, suggesting that STAT3 controls AhR expression (27). As one hallmark of R848-treated APCs is an intense STAT3 activation, we expected an upregulation of AhR. Indeed, we found the induction of AhR in R848-treated APCs when measured by western blot (Figure 5D).

**Figure 6:**
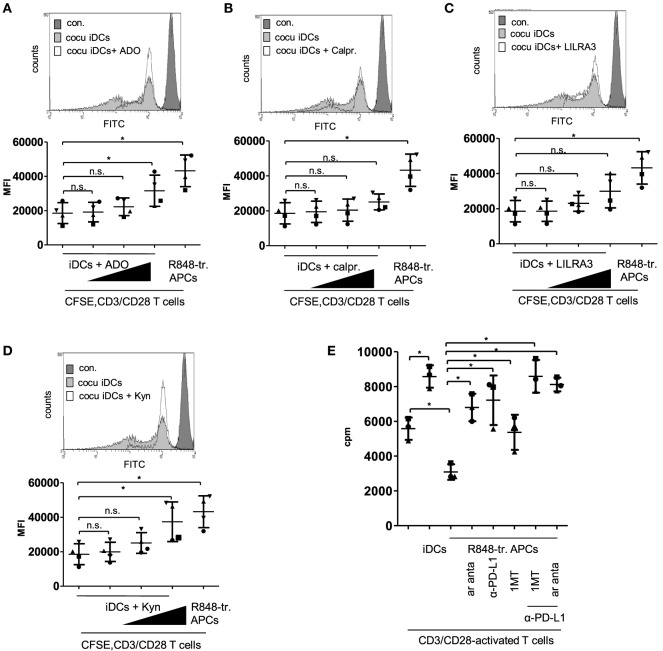
Mimick and inhibition experiments. **(A)** Carboxyfluorescein succinimidyl ester (CFSE)-labeled CD4^+^ T cells were treated with anti-CD3/CD28-coated beads and cocultured for 3 days with immature dendritic cells (iDCs) or R848-treated antigen-presenting cells (APCs). After 3 days, cell divisions were analyzed by determining the FITC signal using a FACScanto. As control non-activated T cells were used, **(B–E)** iDCs were treated with **(B)** adenosine (ADO: 10, 100, 1,000 ng/ml), **(C)** calprotectin (calpr.: 1, 3, 6 µg/ml), **(D)** LILRA3 (1, 10, 100 ng/ml), or **(E)** Kynurenine (Kyn: 100, 1,000, 10,000 ng/ml). After 3 days, allogeneic, CFSE-labeled, CD3/CD28-activated CD4^+^ T cells were added in a ratio of 2:1. Three days later, cell divisions were analyzed by determining the FITC signal using a FACScanto. Top of B-E show FACS histogram overlays (Weasel.jar software), each with the highest concentration of adenosine, Kyn, LILRA3 or calpr. Con. stands for CFSE-stained T cells on day one. Bottom of **(A–D)** shows the according quantitative analyses (mean fluo., mean fluorescence) with mean and SD. **(E)** Mixed lymphocyte reaction (MLR) of CD3/CD28-activated CD4^+^ T cells and iDCs or R848-treated APCs ± 1 µM ADO-receptor antagonist (ar anta), 240 µM indoleamine 2,3-dioxygenase inhibitor 1-methyl-tryptophan (1MT), or 10 µg/ml α-PD-L1 antibody. Proliferation of responder CD4^+^ T cells was measured by [3 H]-thymidine uptake after 96 h of stimulation with allogeneic DCs. Statistic: *p ≤ 0.05 by Mann–Whitney *U* test. MLR was performed in duplicates.

Next we checked the actual impact of the upregulated factors and pathways on the tolerogenic capacity of R848-treated APCs. We started with experiments mimicking the differentiation conditions. We added recombinant ADO, calprotectin, LILRA3, or kynurenine in increasing concentrations to the coculture of iDC and CFSE-labeled CD3/CD28-activated CD4^+^ T cells. Supplementing ADO in the culture medium at a concentration of 100 ng/ml slightly reduced T cell proliferation, whereas 1,000 ng/ml ADO significantly decreased iDC-mediated T cell activation (Figure 6A). Supplemented calprotectin (S100A8/A9) did not even inhibit of T-cell proliferation at a concentration of 6 µg/ml significantly (Figure 6B) and the suppressive effect of LILRA3 was also not significant at the highest chosen concentration (100 ng/ml) (Figure 6C). Lastly, kynurenine inhibited proliferation significantly in a concentration of 10,000 ng/ml and yielded a CFSE signal similar to T cells cocultured with R848-treated APCs (Figure 6D). Therefore, of the four factors tested, kynurenine and ADO mediated the greatest suppression of T cell activation. To verify the actual impact of ADO and IDO in our system, we further performed MLRs with R848-treated APCs and CFSE labeled, activated T cells in culture media containing an ADO-receptor antagonist, the competitive IDO inhibitor 1MT, or anti-PD-L1 antibodies. Previously, we showed that blockade of PD-L1 on R848-treated APCs could partially restore T cell activation (11). Figure 6E shows that the ADO-receptor antagonist (ar anta), anti-PD-L1 (αPD-L1), and 1MT restored R848-treated APC-mediated T cell proliferation significantly. The combination of αPD-L1 with either ar anta or 1MT fully suppressed the inhibitory potency of R848-treated APCs on CD3/CD28-mediated T cell activation.

**Figure 7:**
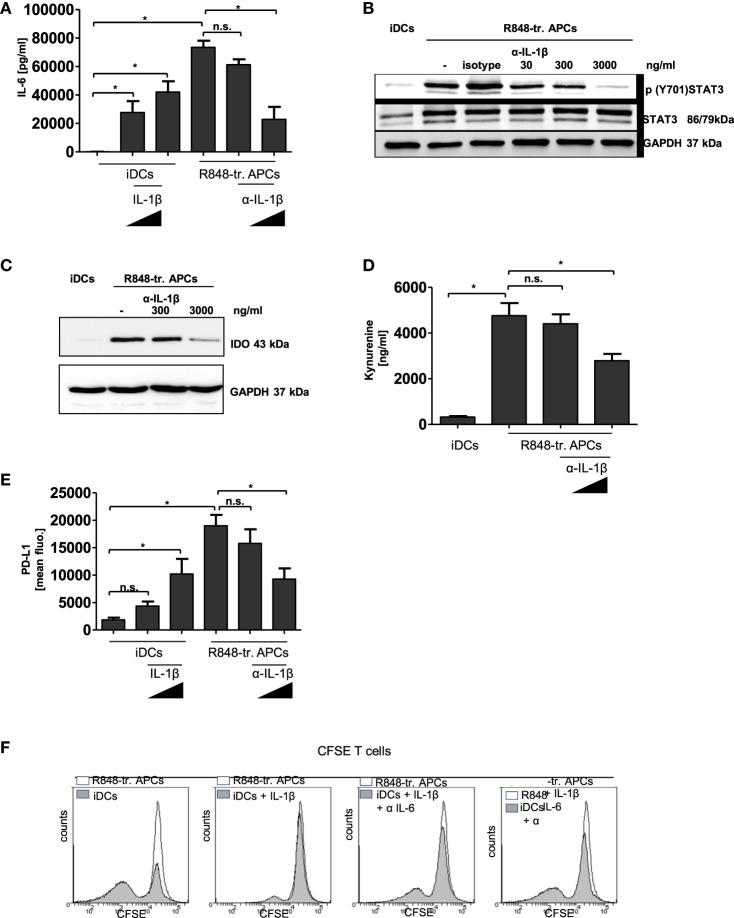
IL-1β in R848-treated antigen-presenting cell (APC) phenotype. Immature dendritic cells (iDCs) were treated with recombinant IL-1β (100 ng/ml, 1,000 ng/ml). R848-treated APCs were treated with an α-IL-1β neutralizing antibody (300 ng/ml, 3,000 ng/ml) or isotype control (3,000 ng/ml). **(A)** IL-6 release was measured by enzyme-linked immunosorbent assay (ELISA). **(B)** Lysates were prepared and adopted in western blot analyses for the detection of *p* (Y701) signal transducer and activator of transcription 3 (STAT3) and total STAT3. GAPDH served as loading control. **(C)** Western blot analyses of indoleamine 2,3-dioxygenase. **(D)** Released Kynurenine was detected in the supernatant of α-IL-1β-treated cells. **(D)** PD-L1 surface expression was detected by flow cytometry. **(A,D,E)** show the mean and SD of three donors. ELISAs are performed in duplicates. **(F)** Monocytes were treated with recombinant IL-1β (3,000 ng/ml) with or without α-IL-6 antibody (1 µg/ml, 10 µg/ml) from the beginning of differentiation into iDCs. After 3 days cells were cocultured with carboxyfluorescein succinimidyl ester (CFSE)-labeled T cells. As control T cells were cocultured with R848-treated APCs. Shown are overlays of flow cytometry data produced with Weasel software.

Finally, we aimed to find the link between the initial hyper-inflammatory and subsequent immunosuppressive phase of R848-treated APCs. In Figure 2C, we show that inhibition of IL-1R signaling during R848-treated APC differentiation yielded a distinct restoration of the activating phenotype. Therefore, we investigated whether an anti-IL-1β-specific antibody would prevent the upregulation of the tolerogenic factors. As IL-6-mediated STAT3 activation is able to induce induction of PD-L1, IDO, and ADO-generating enzymes (11, 28, 29), we first evaluated a possible IL-1β-mediated boost of IL-6 production. To examine this, we either treated R848-treated APCs from the beginning of differentiation with an anti-IL-β antibody or we stimulated iDCs with recombinant IL-1β. As shown in Figure 7A, IL-1β was able to induce production of IL-6 in iDCs. On the other hand, IL-6 released through R848 stimulation could significantly be reduced *via* inhibition of IL-1R-signaling. Since TLR signaling induces IL-6 directly through MAP kinases, we did not expect to fully suppress IL-6 *via* blockade of IL-1β. The diminished amount of IL-6 in the supernatant resulted in a weaker activation of STAT3 (Figure 7B) and, consequently, in the diminished induction of IDO (Figure 7C) and of PD-L1 (Figure 7E). The western blot in Figure 7C shows that blocking of IL-1R-signaling decreased the IDO level in R848 cells in a dose-dependent manner. Figure S4 in Supplementary Material additionally confirms that IDO induction in R848-treated APCs can be inhibited through treatment with a STAT3 inhibitor. In addition, the catalyzed tryptophan degradation to kynurenine was diminished (Figure 7D). Similar results were observed for PD-L1 induction. The FACS analysis in Figure 7E reveals that recombinant IL-1β upregulated PD-L1 on iDCs in a dose-dependent manner. Likewise, the suppression of IL-1R signaling during R848-treated APC differentiation resulted in the downregulation of surface expression of PD-L1. Interestingly, PD-L1 expression seems to be connected to the IDO/kynurenine pathway since treatment with the IDO inhibitor 1MT also downregulated surface expression of PD-L1 (Figure S3 in Supplementary Material). As according to the data IL-1β boosts IL-6 and expression of tolerogenic factors, we further aimed to clarify the impact of IL-6 on IL-1β-mediated reprogramming the APCs. Therefore, we treated monocytes once more with recombinant IL-1β right from the beginning of GM-CSF/IL-4-stimulated differentiation into iDCs. In addition, we blocked IL-6 receptor signaling by adding an anti-IL-6 antibody to the culture. The data in Figure 7F show that increasing concentration of the anti-IL-6 antibody increasingly blocked IL-1β-mediated inhibition of the T cell activating DC phenotype.

## Discussion

Upon infection, peripheral blood monocytes are recruited and activated to boost the inflammatory response. Nevertheless, acute inflammation needs to be controlled and resolved to prevent tissue damage and energy waste. Returning to homeostasis is an active process that may involve several anti-inflammatory and pro-resolving agents, such as nitric oxide and IL-10, produced by regulatory cells, such as M2 macrophages, Tregs, and MDSCs (2, 4). Due to their high plasticity, blood monocytes are best suited to mediate a shift from an inflammatory to resolving response. Well known as precursor cells for immunogenic DCs, monocytes can also differentiate into tolerogenic, suppressive type of immune cells (30–33). This phenomenon can be best observed under hyper-inflammatory conditions apparent in neoplastic and infectious diseases. Interestingly in sepsis, monocytes combine a pro-inflammatory phenotype with anti-microbial yet immunosuppressive and tissue remodeling characteristics (6). This evidences the close connection between immune activation and downregulation. The highly plastic nature of monocytes is well known, albeit a clear determination of the functional phenotype of different subsets and intermediate states is critical to render. Three distinct monocyte phenotypes are distinguished in humans. There are classical monocytes (CD14^+^CD16^−^), intermediate monocytes that are CD14^++^ and CD16^+^, and non-classical monocytes (CD14^dim/−^ CD16^+^) (13, 14, 30). A recent publication challenged the paradigm that the non-classical monocytes are poor TLR responders. Thaler et al. (34) showed in a human *in vivo* approach that CD16^+^ subsets are actually the ones that respond to low doses of endotoxin with high production of IL-6 and IL-8. As CD16^+^ monocytes are known to accumulate in an inflammatory milieu in neoplastic and infectious diseases, this would assume that non-classical cells could sustain hyper-inflammation during infection. Strikingly, a tolerogenic signature is also reported for CD16^+^ monocytes. For example, during viral infection, PD-L1 is strongly upregulated compared to classical monocytes (35, 36). However, the published data on CD16 monocytes regarding co-stimulatory molecules and released cytokines strongly differ. This could be due to varying inflammatory conditions (neoplastic or infectious disease), the lack of a clear boundary between intermediate and non-classical monocytes and different TLR ligands (bacterial, viral). Our study shows that early encounter of peripheral monocytes with PIC mediates differentiation of a T cell-activating phenotype, whereas LPS and most notably R848 induce a pro-inflammatory but T cell suppressing one.

The two opposing faces of R848-treated APCs make perfect sense as the trigger for the onset of tolerance and resolution should be the inflammation itself. Recently, it has become increasingly recognized that, indeed, highly pro-inflammatory mediators, such as IL-1β, TNFα, and IL-6 play a dual role in the immune response as both activators and terminators. For example, Souza et al. (16) showed that during severe intestinal ischemia and reperfusion injury, IL-1β triggered an anti-inflammatory cascade resulting in the production of IL-10. In their study, they associated a blockade of IL-1β signaling with an increase in tissue injury, pro-inflammatory cytokine production, and lethality. Furthermore, polymorphisms of the NLRP3 gene lead to hypo-production of IL-1β that is associated with increased risk of developing Crohn’s disease (17). TLRs are known to mediate transcription of pro-IL-1β through NFκB signaling (37, 38). Enzymatic cleavage of the precursor into the bioactive cytokine can be catalyzed through the caspase-1-containing protein platform inflammasome and also through inflammasome-independent mechanisms (39, 40). In the case of inflammasome-mediated IL-1β activation, another family of PRRs, the NLR are of importance. Interestingly, in contrast to PIC and LPS R848 can directly activate the NLRP3-inflammasome (41). Our data confirm that R848-treated APCs release high amounts of IL-1β and that inhibition of IL-1R signaling mediates differentiation toward are more immunogenic phenotype and inhibits the R848-treated APC-mediated expansion of Tregs. Accordingly, we claim that *in vitro* R848-mediated hyper-inflammation reprograms human blood monocytes toward a tolerogenic, resolving phenotype. The mechanism thereby includes an IL-1β-transduced boost of IL-6 production and endogenous STAT3 activation, which provokes the upregulation of several tolerogenic factors. Whether IL-10 that is released by R848-treated APCs (Figure 1A) is also involved in that mechanism cannot be told by this time of the study. Other immunosuppressive cytokines, such as IL-35 or IL-27, did not turn up in the performed gene array or were only slightly upregulated. Whether they are also involved cannot be confirmed or excluded.

Signal transducer and activator of transcription 3 is known to fulfill a key role in induction of immune-suppressive mechanisms and accomplishes reprogramming of peripheral monocytes into MDSCs (9, 10). In our approach, a combination of STAT3-upregulated PD-L1 together with ADO and IDO were responsible for the observed inhibitory effect on T cell proliferation. All three factors are well known for their immune regulatory function. ADO is a highly anti-inflammatory metabolite of extracellular ATP and generated through CD39 and CD73 in a STAT3-dependent manner (Figure S4B in Supplementary Material). The purine nucleoside is released by Tregs, suppressive B-cells, and MDSCs (42, 43) and modulates the activation status of most immune cells *via* regulation of intracellular cAMP levels (44). IDO is well known for its involvement in immune escape mechanisms of tumors. In the tumor microenvironment, the intracellular enzyme and its metabolites participate in creating an immunosuppressive milieu, for example, by direct inhibition of T cell activation and mediating Tregs (23, 29, 45). Also, during sepsis IDO is strongly upregulated and considered as a tolerogenic factor involved in hyporesponsiveness and immunoparalysis (46). Upregulation of IDO could have two potential consequences during infection. The IDO-catalyzed depletion of tryptophan initially exerts anti-proliferative effects on microbes (47, 48) and, subsequently, after clearance of the infection Trp metabolites such as kynurenine act as an immunosuppressive agent. In addition, PD-L1 is upregulated on peripheral monocytes during the second phase of sepsis (49, 50). Interestingly, IDO and PD-L1 seem to be interconnected, as inhibition of Trp degradation downregulated PD-L1 surface expression (Figure S4 in Supplementary Material). We speculate that the AhR is thereby involved. Accordingly, STAT3-dependent IDO induction catalyzes the degradation of Trp. The generated kynurenine binds and activates AhR, which translocates to the nucleus thereby boosting IL-6 and IL-10 transcription. Therefore, IL-6-mediated STAT3 activation would trigger a positive autocrine signaling loop. In addition, the positive feedback loop could affect ADO generation as well since the Entpd1 (CD39) promoter contains three AhR-responsive elements (51). However, at this point, this is just speculation.

Besides ADO, IDO, and PD-L1, R848-treated APCs additionally show elevated expression levels of S100A8/A9 and LILR family members with proposed immune modulatory function. However, mimic experiments indicate that S100A8/A9 heterodimer calprotectin and LILRA3 are less important for the direct inhibition of T cell proliferation (Figures 6B,C) as the supernatant of R848-treated APCs do not contain effective concentrations of the factors. One can speculate that they play a role as endogenous regulators of the tolerogenic R848-treated cell phenotype. Indeed, overexpression of inhibitory LILRs is a feature of tolerogenic myeloid DCs. By binding their highest affinity self-ligand HLA-G (soluble or cell-bound), classical inhibitory LILR family members modulate intracellular calcium levels *via* their immunoreceptor tyrosine-based inhibitory domains. Thereby they induce and maintain a suppressive APC phenotype (52, 53). The modulatory role of LILRA3 is less clear. It could affect cells by acting as an antagonist for activating LILRs. In addition, calprotectin is reported to be involved in the generation of immunosuppresive cells (54).

During normal differentiation of myeloid precursors to DCs and macrophages S100A8 and S100A9 are downregulated. However, the prolonged STAT3 activation in cells of the tumor microenvironment preserves their production, thus resulting in differentiation and accumulation of MDSCs (55, 56). Our data reveal that R848-treated APCs have a high surface expression of inhibitory LILRB1 and LILRB2 and supernatants contain increased amounts of HLAG, LILRA3, and calprotectin. Nevertheless, further experiments are necessary to clarify their actual impact on generation of the resolving phenotype.

Taken together, our *in vitro* study emphasizes the high plasticity of the myeloid lineage that enables an inflammation-induced and STAT3-mediated shift to a tolerogenic APC phenotype. One could speculate that during infection peripheral monocytes are stimulated to mediate immunity and simultaneously prime themselves for subsequent downregulation of the response after clearance of the infection. Although our *in vitro* system naturally cannot constitute the complex scenario of an infection, the presented data suggest a crucial role for peripheral plastic monocytes in guiding the immune response.

## Ethics Statement

This study was carried out in accordance with the recommendations of the ethics committee of the Medizinische Fakultät Heidelberg with written informed consent from all subjects. All subjects gave written informed consent in accordance with the Declaration of Helsinki. The study (taking of blood samples from healthy donors and treatment of blood leukocytes with microbial stimuli) was reviewed and approved by the ethics committee of Medizinische Fakultät Heidelberg.

## Author Contributions

KH and DH designed the study with essential input from KG, M-EE, SW, and KB. DH wrote the final manuscript. KG, ME, SW, AS, DS, ZM, VO, and KB performed the experiments. All authors read the manuscript and discussed the results.

## Conflict of Interest Statement

The authors declare that the research was conducted in the absence of any commercial or financial relationships that could be construed as a potential conflict of interest. The reviewer, RD, and handling editor declared their shared affiliation, and the handling editor states that the process nevertheless met the standards of a fair and objective review.

## Footnotes

^1^https://www.dkfz.de/gpcf/illumina_beadchips.html.

^2^http://chipster.csc.fi/

^3^https://www.ncbi.nlm.nih.gov/geo/query/acc.cgi?acc=GSE98480.
