# Supplementary material for: IL-1β As Mediator of Resolution That Reprograms Human Peripheral Monocytes toward a Suppressive Phenotype
Source: Front Immunol. 2017 Aug 3;8:899. doi: 10.3389/fimmu.2017.00899 (PMC5540955; doi:10.3389/fimmu.2017.00899)
Supplement: Supplementary file 1 [file Presentation_1.PDF]

Supplementary Material

IL-1β as mediator of resolution that reprograms human peripheral monocytes towards a suppressive phenotype

Katharina Giesbrecht[1,2+,] Mariel-Esther Eberle[1+], Sabine J. Wölfle[1], Delal Sahin[1], Aline Sähr[1], Valerie Oberhardt[1], Zach Menne[1], Konrad A. Bode[1], Klaus Heeg[1,2], Dagmar Hildebrand[1]*

[1]Medical Microbiology and Hygiene, Centre for Infectious Diseases, University Hospital Heidelberg, Heidelberg, Germany

[2] DZIF German Center for Infection Research

[+] boths authors contributed equally

*** Correspondence:**

Dagmar Hildebrand

dagmar.hildebrand@med.uni-heidelberg.de

Supplementary Methods

2D Differential In-Gel Analysis (DIGE)

1x10^7 cells were lysed in urea lysis buffer ([9.5 M urea, 1% (w/v) dithiothreitol (DTT), 2% (w/v) CHAPS, 2% (v/v) carrier ampholytes (pH 3–10) and 10 mM Pefabloc® proteinase inhibitor). Fluorescence labelling of the cell lysates was carried out according to the manufacturer's protocol for minimal CyDye labelling (GE Healthcare). 50 µg protein of each sample were labelled with 200 pmol of amine reactive cyanine dyes, Cy3 or Cy5 and 50 µg protein of a mixture of both samples with Cy2 (GE Healthcare). The pooled (Cy2, Cy3 and Cy5) samples were cup loaded on a 24 cm IPG strips, pH 3–11. Isoelectric focusing was performed using the IPGphor II isoelectric focusing system for a total of 45 kVh at 20℃. Thereafter the IPG gel was incubated in equilibration buffer (50 mM Tris HCl, pH 8.8, 6 M urea, 30% glycerol, 2% SDS, 0.002% bromophenol blue) supplemented with 0.5% DTT for 15 min, followed by 4.5% iodoacetamide in fresh equilibration buffer for an additional 15 min. The strip was immediately applied on a 8% – 12% SDS Gel (25 x 20 cm; Ettan Dalt Six, GE Healthcare and the gels were run at 20 °C with a constant current of 8 mA/gel for 1 h, followed by 16 mA/gel until the end of the run. After the second dimension, the gels were scanned on a Typhoon Trio imager (GE Healthcare) with 100 µm resolution. Image analysis was performed using the Batch Processor module of the DeCyder 7 software (GE Healthcare), following the manufacturer's recommendations. The spots were quantified on the basis of relative volume. For identification of the proteins within a spot of interest, spots are picked after gel staining with Deep Purple with a Spot picker.

Peptide mass fingerprinting (PMF):

Identification of SDS-PAGE-separated proteins was performed on reduced (DTT or TCEP, 10 mM in 50 mM ammoniumbicarbonate ("ABC buffer"), pH 7.5, 50°C, 30 min), alkylated (50 mM iodoacetamide in ABC buffer, 1h, RT), and trypsin-digested (15 ng/ul, 37° C, o/n) samples. Proteolytic digests were loaded using a nano-HPLC (Proxeon easy-nLC) on reverse phase columns (trapping column: particle size 5 µm, C18, L=20 mm; analytical column: particle size 3µm, C18, L=15cm; NanoSeparations, Nieuwkoop, The Netherlands), and eluted in gradients of water (0.1 % formic acid, buffer A) and acetonitrile (0.1 % formic acid, buffer B) Typically, gradients were ramped from 5 % to 55 % B in 50 minutes at flowrates of 300 nl/min. Peptides eluting from the column were ionised online using a Bruker Apollo ESI-source with a nanoSprayer emitter and analysed in a quadrupole time-of-flight mass spectrometer (Bruker maXis). Mass spectra were acquired over the mass range 50-2200 m/z, and sequence information was acquired by computer-controlled, data-dependent automated switching to MS/MS mode using collision energies based on mass and charge state of the candidate ions. The data sets were processed using a standard proteomics script with the software Bruker DataAnalysis 4.0 Service Pack 1 Build 253 and exported as mascot generic files. Spectra were internally recalibrated on autoproteolytic trypsin fragments when applicable. Proteins were identified by matching the derived mass lists against the NCBI nr

database (downloaded from http://www.ncbi.nlm.nih.gov/) on a local Mascot server (Matrix Science, UK). In general, a mass tolerance ± 0.05 Da for parent ion and fragment spectra, two missed cleavages, oxidation of Met and fixed modification of carbamidomethyl cysteine were selected as matching parameters in the search program (p = 0.05, 2 peptides per protein).

Determination of Indolamin-2,3-dioxygenase activity

For the detection of the enzymatic activity of IDO, kynurenine was measured in the supernatants of iDCs and R848-treated APCs +/- 240µM 1MT (1h prior to stimulation and again after 2 days) after 3 days of culture. The protocol was adapted to the manufacturers instruction (Universität Ulm, Transplantationsforschung, Ulm, Germany). In brief, 150 µl cell culture supernatant was supplemented with 100 µl Trichloric acid (30%) for protein precipitation. After centrifugation, supernatant was transferred into 96-well plate format and incubated at 50°C for 30 min. Absorbance was measured at 492nm with a reference wavelength of 690 nm using a photometer (SUNRISE Absorbance reader, Tecan, Salzburg, Austria). Kynurenine concentrations were calculated with the Magellan V 5.0 software (Tecan, Salzburg, Austria).

Supplementary Fig.1

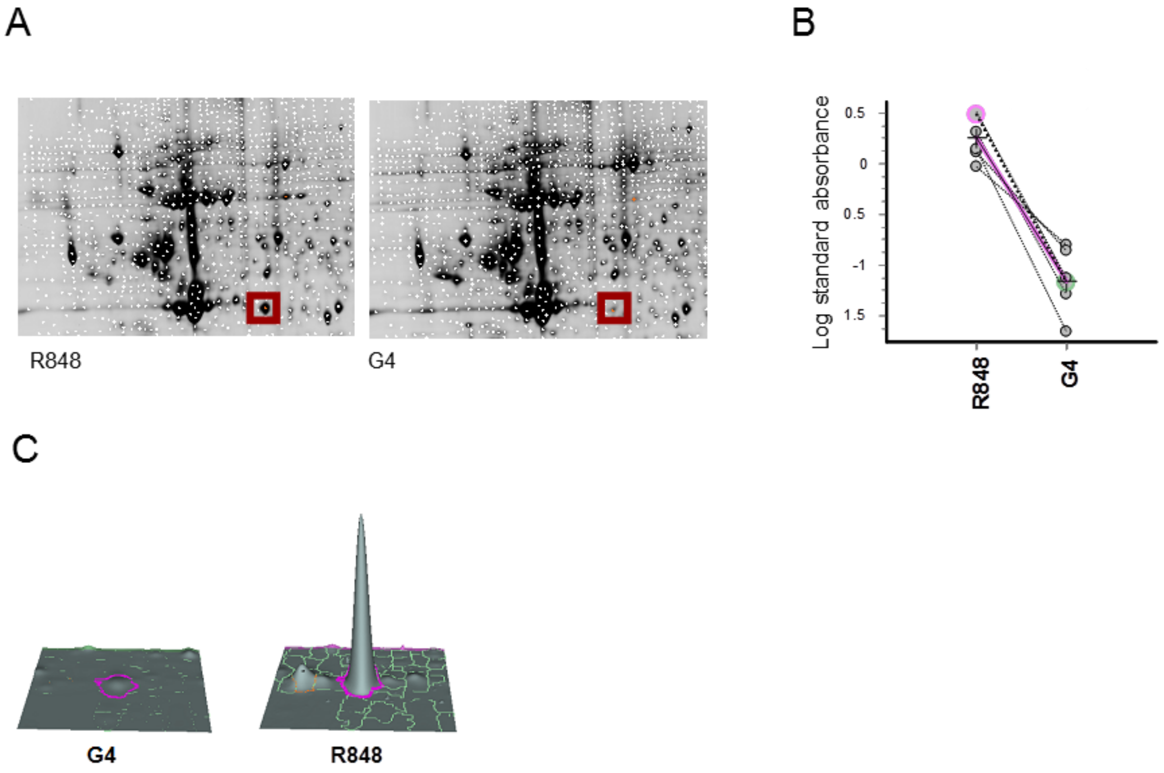

2D Differential In-Gel Analysis (DIGE) of R848-treated APCs in comparison to iDCs.

Whole cell lysates of iDCs , R848-treated APCs and a mixture of both were labeled separately with CyDye DIGE fluors. A mixture of equal protein amounts of the three labeled samples were separated simultaneously on a 2D gel. (A) Representative gel image (cropped) for the labeling of R848-treated APCs (left,R848) and labeling of the iDCs ((right, G4). (B) Expression profile for the marked protein spot (pro-IL-1β) of six independent experiments, (C) 3D view of these protein spots obtained by BVA module of DeCyder software. LCMS Analyses of the protein spot identified the spot as pro-IL-1 β.

Supplementary Fig.2

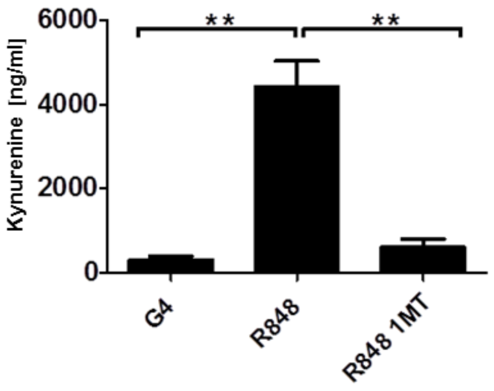

Supernatant of iDCs (G4) and R848-treated APCs (R848) +/- 100µM IDO inhibitor 1-methyl tryptophan (1MT) were analyzed for kynurenine. Shown is the mean and standard deviation of three donors. Statistic: * p</=0.05 by Mann–Whitney U test.

Supplementary Fig.3

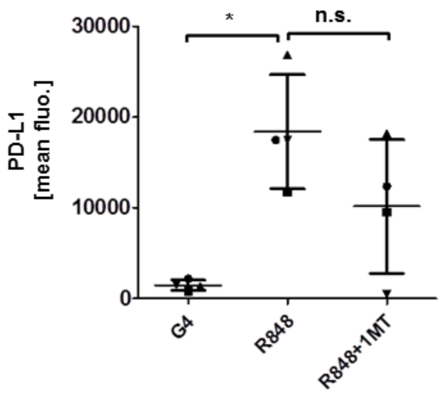

Cells (iDCs = G4, R848-treated APCs = R848) were treated with 100µM 1MT. After three days PD-L1 was quantified by antibody-staining and flow cytometry. Each symbol depicts one donor. Additionally shown is the mean and standard deviation. Statistic: * p</=0.05 by Mann–Whitney U test.

Supplementary Fig.4

A

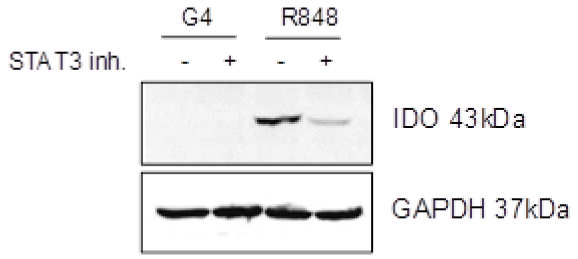

B

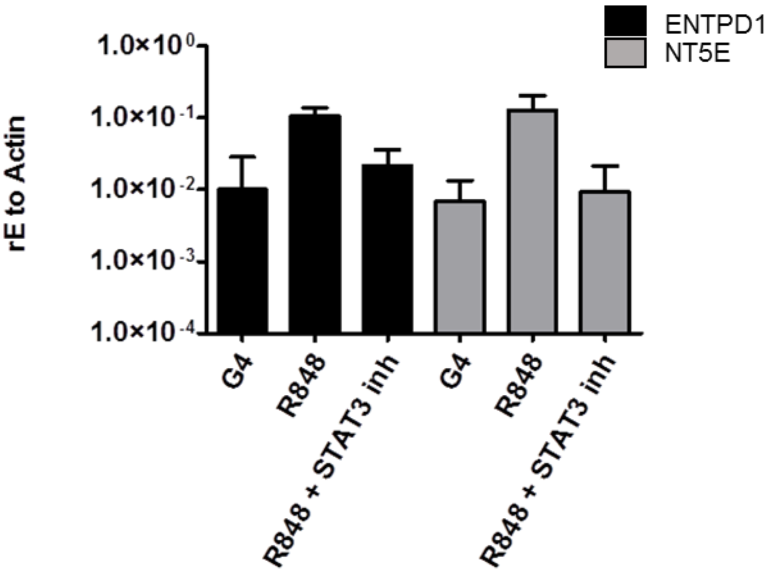

Dependency of tolerogenic factors in STAT3. Isolated CD14+ monocytes were pre-treated for 2 h with 200nM STAT3 inhibitor JSI-124 (Calbiochem Schwalbach, Germany) and then stimulated with GM-CSF and IL-4 (iDCs = G4) or GM-CSF, IL-4 and R848 (R848-treated APCs = R848) for three days. A) For western blot analysis equal amounts of protein lysates were blotted (GAPDH as loading control) and probed with antibodies against IDO. B) RNA was isolated and cDNA produced. Induction of ENTPD1 (gene encoding CD39) and NT5E (gene encoding CD73) was determined by rt PCR using sequence-specific primer and SYBR Green Master mix. Results were normalized against β-Actin. Primer sequence can be found in the material and methods section of the main text.
